# Stage 1 Registered Report. Interventions for improving the design and conduct of scientific research: A scoping review

**DOI:** 10.3310/nihropenres.13252.1

**Published:** 2022-02-01

**Authors:** Andrew Mott, Catriona McDaid, Catherine Hewitt, Jamie J Kirkham

**Affiliations:** 1 York Trials Unit, University of York, UK, York, North Yorkshire, YO10 5DD, UK; 2 Centre for Biostatistics, The University of Manchester, Manchester, Manchester, M13 9PL, UK

**Keywords:** Meta-Research, Research-on-Research, Research Design, Research Conduct, Research Waste, Scoping Review

## Abstract

**Background:**

Research waste is prevalent in many scientific fields despite a number of initiatives to improve research practices. Interventions to improve practice are often implemented without evaluating their effectiveness. It is therefore important to identify the interventions that have been evaluated, assess how they have been evaluated and to identify areas where further research is required.

**Methods:**

A scoping review will be undertaken to assess what interventions, aimed at researchers or research teams, to improve research design and conduct have been evaluated. This review will also consider when in the research pathway these interventions are implemented; what aspects of research design or conduct are being targeted; and who is implementing these interventions.

Interventions which aim to improve the design or conduct of research will be eligible for inclusion. The review will not include interventions aimed at hypothetical research projects or interventions implemented without evaluation.

The following sources will be searched: MEDLINE, EMBASE, ERIC, HMIC, EconLit, Social Policy and Practice, ProQuest theses, and MetaArXiv. Hand searching of references and citations of included studies will also be undertaken. Searches will be limited to articles published in the last 10 years.

Data extraction will be completed using a data extraction template developed for this review.

Results will be tabulated by type of intervention, research stage, and outcome. A narrative review will also be provided addressing each of the objectives.

## Plain language summary

It has been shown that due to the poor way in which some research is done the results either cannot be trusted or are not useful. Examples of this include: not publishing research that is completed so the results cannot be used by others; using outdated or unreliable research methods; measuring outcomes that are not useful in the real world or not important to patients; having too small a sample of participants in a study to get a reliable answer to the research question. This means that the money that has been spent on the research and the time that any participants have given to that research has been wasted.

There have been a number of initiatives to try and stop this happening. It is important to evaluate these to test whether they actually work. Some initiatives that have been evaluated include: reminders to publish research once it is complete, providing researchers with tools for better research design and public recognition for good research practices.

We intend to search for and summarise all the initiatives that have already been tested. To do this we will do a comprehensive search for all studies that have tested these initiatives. By doing this we will be able to see what further initiatives could be developed and how we can better test them.

## Introduction

Estimates have suggested that as much as 85% of research is waste ^1^. With over $100 billion spent annually on medical research this amount of wastage is extremely costly. Glaziou and Chalmers ^1^ identified four overarching stages in the research process where waste can occur: question and priority setting; appropriate design and methods; availability of results; unbiased and usability of results.

Solutions to all these aspects of research waste have been suggested including the development of guidelines, policies, laws, incentives, and accepted standards ^2, 3^. However, research waste persists despite the development of better standards and practice, with the coronavirus disease 2019 (COVID-19) pandemic exacerbating poor practices due to the speed at which research was required ^3, 4^.

Hardwicke *et al.* ^5^ proposed a framework for improving research practice that has four stages: 1) identify problems; 2) investigate problems; 3) develop solutions; 4) evaluate solutions. They note that many solutions are developed without any consideration for evaluation and that unintended consequences or lack of anticipated benefits may go undetected. They suggest that as a result of this many existing evaluations of these interventions are retrospective observational studies. Previous reviews in this area have looked at individual interventions or individual problems, such as interventions to improve the reporting of research ^6^, incentives for data sharing ^7^, and interventions for increasing the publication of research ^8^. 

There has however been no overarching assessment of interventions to improve the design and conduct of research. Therefore, the aim of this scoping review is to map the evaluations of interventions which aim to improve the design or conduct of research. We will use this review to identify knowledge gaps and to assess how this type of research could be improved. Some examples of interventions that we were aware of prior to starting this research include: awarding badges for data sharing ^9^; legal requirements for publication ^10^; funder recommendations for design choices ^11^; email reminders for increasing publication ^12^; providing training and tools to researchers to allow them to design better studies ^13^.

### Objectives

The aims of this scoping review are to address the below questions.

- What interventions, aimed at researchers or research teams, to improve the design and conduct of research have been evaluated?
  ○ Where in the research pathway are these interventions implemented?
  ○ What specific aspects of research are being targeted?
  ○ Who is implementing these interventions? (funders/ethics committees/research institutions/publishers/etc.)

## Protocol

### Registration

The methodology of this scoping review will be registered with the Open Science Framework registry following peer review of the stage 1 registered report publication and before any searches are undertaken.

### Eligibility criteria

This review will include any evaluations of interventions that aim to improve the design or conduct of scientific research by targeting researchers or research teams.

***Population*.** Researchers or research teams undertaking or developing research projects. This will not include studies where the intervention is aimed at those participating in research (such as Studies Within A Trial).

***Concepts*.** For this review, any intervention will be considered such as policies, laws, and guidelines provided there has been a reported evaluation of the effectiveness of the intervention.

Interventions aimed at any aspect of design and conduct will be included from initial design (e.g. question setting, protocol development, etc.), through undertaking the research (e.g. registration, changes to research design, etc.), and publishing (e.g. timely publication, reporting standards, data sharing, etc.).

***Context*.** This review will include any interventions aimed at scientific research. This review will not include interventions designed to improve hypothetical research projects, such as those that a research student may develop for a research methods course.

There will be no other limitation on the context that the interventions are applied in.

***Types of Studies*.** This review will include any quantitative or qualitative evaluation of interventions but will not include interventions that have not been evaluated.

### Information sources and searching strategy

Literature searches of the following electronic databases will be made: MEDLINE, EMBASE, EconLit, ERIC, and Social Policy and Practice. The following grey literature sources will be searched: HMIC, ProQuest Dissertations and Theses Global and all articles submitted to the MetaArXiv preprint server.

Searches were developed with previously identified studies using the Yale Mesh Analyser and the PubReminer tools and guidance from an information specialist. The full search strategy is available as extended data ^14^. Hand searching will be undertaken of references within and citations to included articles to identify any further relevant articles.

To aid the identification of relevant research we will contact authors, researchers and other stakeholders (funders, publishers, research organisations, etc.).

No limits will be applied relating to language or source of information. Where translation is required, Google Translate will be used. The searches will be limited to the last 10 years.

### Selection of sources of evidence

All records identified will be de-duplicated using Endnote v20 then imported into Covidence Systematic Review software. Free alternatives that perform similar functions are also available.

A two-stage screening process will then be used, first screening the titles and abstracts of all identified studies and then screening the full-texts of any that appear eligible at the first stage. Both stages will be completed independently by two researchers with any disagreements resolved by discussion.

This process will be piloted initially using a random selection of 25 items and will be continued until 75% or higher agreement is achieved. 

### Data charting process

Data extraction will be completed using a data extraction template. This process will be done by one researcher with a second independently checking with any disagreement being resolved through discussion. This process will be piloted on a small number of initial studies to ensure consistency and that all pertinent data is being captured in the data extraction template.

***Data items*.** The below items of data are the initial items to be extracted, any further items identified during the extraction process will be reported.

- Author(s) or Organisation(s)
- Year of publication
- Country
- Population
- Sample size

- Evaluation Methodology
  ○ Evaluating organisation

- Intervention details
  ○ Intervention description & rationale
  ○ Intervention aim
  ○ Duration of intervention
  ○ Who is implementing the intervention
  ○ Where in the research pathway is this implemented

- Comparator (if applicable)

- Outcomes
  ○ Outcomes measured
  ○ Timeframe of outcomes

- Author’s conclusion as verbatim text

As well as this a type of intervention will be assigned according to the intervention function categories in the behaviour change wheel framework as below ^15^.

- Education - Knowledge of practices
- Persuasion - Communication for improving actions
- Incentivisation - Expectation of reward
- Coercion - Expectation of punishment
- Training - Imparting skills
- Restriction - Rules or regulation
- Environmental restructuring - Changing physical or social context
- Modelling - Showing example of good practice
- Enablement - Reducing barriers/increasing means to practice
- Other - Anything not captured by framework

### Analysis and synthesis of results

Results of the searching process will be presented in a flowchart as per the PRISMA-SCR guidance ^16^.

Extracted data will be presented in tables, the following items will be tabulated and this will be done separately for aspects of design and conduct.

- Type of Intervention as per behaviour change wheel
- Stage of research pathway
- Outcome

Any further tables or visualisations of the data that will be informative for the objectives of this review will be considered at the time of analysis. A narrative review will be presented alongside this addressing each of the review objectives which will summarise the key findings and any gaps in the research. Whilst no formal assessment of research quality will be made in this review the research methodology used to evaluate these interventions will be presented and discussed.

## Discussion

This scoping review will summarise the interventions targeted at researchers and research teams that have been evaluated for improving the design and conduct of scientific research. We will assess the characteristics and the expected outcomes of these interventions and look at when in the research pathway these are utilised. We then intend to use the results of this review to identify any gaps in this research and to inform the development and testing of an intervention.

### Study status

No data collection has been started for this study. Searches and data collection will begin following in-principle acceptance of the stage 1 registered report. Completion is anticipated to be by October 2022.

### Dissemination

Following completion of the synthesis, the results of this project will be submitted for publication as a stage 2 registered report. Data related to the results will be made available.

## Ethics

As this research is a literature-based review no ethical approval is required.

## Data availability

### Underlying data

No underlying data are associated with this article.

### Extended data

Open Science Framework: Interventions for improving the design and conduct of scientific research: A scoping review. https://doi.org/10.17605/OSF.IO/257XV

This project contains the following files:

- Scoping Review - Full Search Strategy.pdf
- Protocol - PRISMA- SCR checklist.pdf

Data are available under the terms of the Creative Commons Zero "No rights reserved" data waiver (CC0 1.0 Public domain dedication).
