# Supplementary material for: Interventions for improving the design and conduct of scientific research: A scoping review protocol
Source: NIHR Open Res. 2022 Jun 14;2:4. Originally published 2022 Feb 1. [Version 2] doi: 10.3310/nihropenres.13252.2 (PMC10593266; doi:10.3310/nihropenres.13252.2)
Supplement: Peer review history

# Reviewer report: Reviewer response for version 2

**DOI:** 10.3310/nihropenres.14405.r29021

**Published:** 2023-02-27

**Authors:** Olalekan Lee Aiyegbusi

**Affiliations:** 1 Centre for Patient Reported Outcomes Research, Institute of Applied Health Research, University of Birmingham, Birmingham, UK

This is a very relevant study which could potentially assist with the minimisation of research waste. Well written protocol.

 Comments

- Page 2 of methods: “A narrative review will also be provided…” this is a bit confusing. Do you mean ‘narrative synthesis’? The phrase ‘narrative review’ was used again in the final paragraph of the section on Analysis and synthesis of results (page 5). A narrative review is a different type of review – so please check and revise.
- Page 4: I understand that the focus of the review is on evaluated interventions. However, it would be helpful if this review captures what is out there whether they are evaluated or not. Such information itself is important. These unevaluated interventions could just be mentioned in a Table without having to provide extra details. 

Is the study design appropriate for the research question?

Yes

Is the rationale for, and objectives of, the study clearly described?

Yes

Are sufficient details of the methods provided to allow replication by others?

Yes

Are the datasets clearly presented in a useable and accessible format?

Not applicable

Reviewer Expertise:

Evidence synthesis including systematic reviews, scoping and narrative reviews

I confirm that I have read this submission and believe that I have an appropriate level of expertise to confirm that it is of an acceptable scientific standard.

# Reviewer report: Reviewer response for version 2

**DOI:** 10.3310/nihropenres.14405.r28683

**Published:** 2022-06-28

**Authors:** Anisa Rowhani-Farid

**Affiliations:** 1 Department of Pharmaceutical Health Services Research, University of Maryland School of Pharmacy, Baltimore, MD, USA

The authors have addressed the questions that I had raised in my original review. The only suggestion I have is that it would be helpful if they could indicate that they will also be including pre-prints in their study sample.

Is the study design appropriate for the research question?

Yes

Have the authors pre-specified sufficient outcome-neutral tests for ensuring that the results obtained can test the stated hypotheses, including positive controls and quality checks?

Partly

Is the rationale for, and objectives of, the study clearly described?

Yes

Are sufficient details of the methods provided to allow replication by others?

Partly

Are the datasets clearly presented in a useable and accessible format?

Not applicable

Reviewer Expertise:

Meta-research (with a focus on incentives, reproducibility, reporting)

I confirm that I have read this submission and believe that I have an appropriate level of expertise to confirm that it is of an acceptable scientific standard.

# Reviewer report: Reviewer response for version 1

**DOI:** 10.3310/nihropenres.14368.r28468

**Published:** 2022-03-01

**Authors:** Anisa Rowhani-Farid

**Affiliations:** 1 Department of Pharmaceutical Health Services Research, University of Maryland School of Pharmacy, Baltimore, MD, USA

This study has an interesting and unique study question – ‘what interventions, aimed at researchers or research teams, to improve the design and conduct of research have been evaluated?’

 My only feedback and why I checked ‘partly’ for questions 1 and 4 above is that they have not defined what they mean by ‘interventions’ and by ‘evaluated’. Therefore, it would be good if they could clarify what study types will fit the inclusion criteria (and the exclusion criteria). For instance, will they be including observational studies of interventions? Or will they limit their search to randomized controlled trials of interventions? Further, what does it mean for an intervention to be evaluated? Do they mean ‘tested using the gold-standard’? There are plenty of interventions to improve research conduct but there are few randomized controlled trials. As such, more clarification/definitions will be very helpful. On a related note, is there a reason Scopus, Web of Science, Google Scholar and medRxiv were not included in the list of databases that will be searched?

Is the study design appropriate for the research question?

Yes

Have the authors pre-specified sufficient outcome-neutral tests for ensuring that the results obtained can test the stated hypotheses, including positive controls and quality checks?

Partly

Is the rationale for, and objectives of, the study clearly described?

Yes

Are sufficient details of the methods provided to allow replication by others?

Partly

Are the datasets clearly presented in a useable and accessible format?

Not applicable

Reviewer Expertise:

Meta-research (with a focus on incentives, reproducibility, reporting)

I confirm that I have read this submission and believe that I have an appropriate level of expertise to confirm that it is of an acceptable scientific standard, however I have significant reservations, as outlined above.

# Response

**Published:** 2022-06-08

**Authors:** Andrew Mott

**Affiliations:** University of York, UK, UK

Thank you for reviewing our article, we hope that the our responses outlined below address your points.

*This study has an interesting and unique study question – ‘what interventions, aimed at researchers or research teams, to improve the design and conduct of research have been evaluated?’*

*My only feedback and why I checked ‘partly’ for questions 1 and 4 above is that they have not defined what they mean by ‘interventions’ and by ‘evaluated’.*

 Further clarification has been provided regarding what is meant by an intervention in the "Concepts" section of the Eligibility Criteria and further on evaluated in the types of studies section.

 "For this review, any intervention will be considered, **for the purposes of this review an intervention will be defined as per the behaviour change wheel framework to be any coordinated sets of activities designed to change specified behaviour patterns 15**. 

**Therefore, it would be good if they could clarify what study types will fit the inclusion criteria (and the exclusion criteria). * For instance, will they be including observational studies of interventions? Or will they limit their search to randomized controlled trials of interventions? Further, what does it mean for an intervention to be evaluated? Do they mean ‘tested using the gold-standard’?* *There are plenty of interventions to improve research conduct but there are few randomized controlled trials. As such, more clarification/definitions will be very helpful.*

 We agree that there will be few trials and anticipate that there many of the studies will be retrospective observational designs. We therefore intend to include any quantitative or qualitative assessments of these interventions and to summarise the methods used to evaluate these interventions to date. To clarify this, additional text has been added to the "types of studies" section of the Eligibility Criteria as below.

 "This review will include any quantitative or qualitative evaluation of interventions but will not include interventions that have not been evaluated. **No limitations will be made by study type and will include observational, experimental/quasi-experimental, and qualitative designs as this review intends to summarise the methods used to evaluate these interventions."**

*On a related note, is there a reason Scopus, Web of Science, Google Scholar and medRxiv were not included in the list of databases that will be searched?*

 We developed the searches with an information specialist and tested our key search terms on a number of databases. We therefore feel that our current strategy has the most suitable balance of coverage of the literature without the need to search too many databases. I provide our reasoning for each of these sources below.

 Both MedRXIV and BioRXIV are now searchable through EMBASE which we intend to search so we should capture any appropriate references from these sources within our current strategy. Further details provided here: https://library.medschl.cam.ac.uk/blog/2021/11/medrxiv-and-biorxiv-now-searchable-through-embase/

 Whilst Web of Science is only a platform to search databases we did consider searching the Science Citation Index Expanded database within WOS and SCOPUS but found that many of the results these databases provided for key search terms did not provide a suitable balance of coverage to the number of search results, given the results from other databases chosen.

 We do not feel that Google Scholar would be appropriate for this review. Google scholar is subject to personalisation bias and has no formal method for providing all results of a search or repeating a search in a reproducible manner. Some further details are provided here: https://dx.doi.org/10.5210/ojphi.v5i2.4623
